# A Resume of the Work of the Physicians’ League during Its First Decade1President’s address, read before the Physicians’ League, May 26, 1902.

**Published:** 1902-08

**Authors:** Electa B. Whipple

**Affiliations:** Buffalo, N. Y., 491 Porter Avenue.


### A Resume of the Work of the Physicians' League During its First Decade.[1]

By ELECTA B. WHIPPLE, A. M., M. D., Buffalo, N. Y.

THERE comes a period in the life of every individual when it is beneficial to call a halt, make a review and take an inventory. The growth and advancement in medicine are marked by decades, and each decade has its characteristics. It seems fitting after the ten years' existence of our modest league, to cast up our accounts and approximately ascertain where we stand. The excuse for our existence ten years ago was founded upon the feeling of good fellowship, kindred ambition and the desire as associates "in the great workshop to join in the emulation of each other's attainments, enjoy our grand delusions, criticise each other's ideals and compare results."

We are now ten years old and have passed the experimental age—which all girls have—of trying on mother's (Alma Mater's) dress and proudly watching its long train as it sweeps the street far behind us. But we are now looking forward to the time when with impunity we may don our own trained dress, have our "coming out" and stand rank and file for "luminous comparison" with associations of older growth.

1. President's address, read before the Physicians' League, May 26, 1902.

In the fall of 1892, Drs. Jane W. Carroll, Mary I. Denton, Mary T. Green, Lillian C. Randall and Electa B. Whipple decided to form themselves into an organisation, known as the Medical League—to meet once a month from house to house. In January, 1894, Drs. Helene Kuhlman and Maud J. Frye became members, and in 1895, for good reason, the name was changed from Medical League to Physicians' League.

Ours is essentially a woman's league, although at the time of its organisation it was resolved that our brothers in the profession should not be precluded from becoming members. However, as members come into the league upon invitation only, our womanly modesty will likely continue to prevent us from changing the personnel of the league. From the initial membership of 5, the league has gradually added to its members until now 21 names—1 having resigned upon leaving the city—are registered, with 16 resident members.

There have been 97 meetings of the league and 87 papers presented and discussed since January, 1894—the first date of which we have record. Seventy-eight of the papers have been by members and 11 by invited guests, 8 of whom were women, viz.: Mesdames Mankell and Milanowski, of Buffalo, and Drs. Potter, Ballentine, Dickinson and Baldwin, of Rochester and Adams of La Crosse, Wisconsin. Three were by Drs. Mann, Wende and Gregory, of Buffalo. Thirty-three of the 78 papers have been on internal medicine, viz.: Cystitis, Parenchymatous nephritis, Diabetes mellitus, Chronic constipation, Pertussis, Exophthalmic goitre, Infectious diseases of childhood, Typhoid fever, Autointoxication, Bowel lesions, Tachycardia, Variola and varioloid, Epilepsy, Chorea, Scarlet Fever, Cerebral softening, Fissure of the anus, Gout, Hot-air treatment of rheumatism, Lithemia, Syphilis, Pyorrhea alveolis, Leprosy, Neurasthenia, Hysteria, Infantile Scurvy, Schott's treatment of heart disease, Sea-sickness and Cyclic vomiting. Twenty papers treated of gynecological and obstetrical subjects, viz.: Prolapsus uteri in the aged, Confinement, Early diagnosis of uterine cancer, Cardiac diseases complicating pregnancy, The infant and its food, Licensing midwives, Curettement and dilatation, Discharge from the uterine mucosa, Hydrocele in women (translation), Hydramnios, Intermenstrual phenomena, Use of the pessary in retro-displacements of the uterus, The bicycle saddle, Retro-deviations of the uterus, Dysmenorrhea in young girls, Amenorrhea, Helps in the treatment of version and prolapsus uteri, Treatment of endometritis, Antisepsis in obstetrics and Malaria complicating the puerperium.

Ten papers pertained to materia medica, viz.: Salol, Ichthyol, Protonuclein and allied drugs, Animal extracts (thyroid, suprarenal capsules, bone marrow, etc.) Guaiacol, Lysol, Antipyrin, Orthoform, Some uses of Brewer's yeast, Patent and proprietary remedies.

Two papers on hygiene, viz.: School hygiene and The necessity of medical inspection in the public schools. Three were upon insanity, viz.: Insanity of childhood, Paresis and Dementia precox. Two papers given were upon bacteriology, viz.: The bacteriology of milk, and A study of the bacillus of diphtheria. One treated of electro-therapy and five papers were upon miscellaneous topics, viz.: Post gradute work at Johns Hopkins, Some personal observations in European clinics, Library of the Physicians' League, The Paquelin cautery and Babies' ward of the Erie County hospital.

Madame Mankell gave a paper on Medical gymnastics and massage, and Mrs. Milanowski one upon, The social condition of women in Germany. Dr. Marion Craig Potter, of Rochester, presented a paper on Retro-deviations of the uterus and treatment. Dr. Ballentine, of the State Hospital, Rochester, on Acute delirious mania. Dr. Mary Dickinson, of Rochester, on Diseases of the nose, throat and ear and their relation to other diseases. Dr. Baldwin, of Rochester, Early repair of the cervix and perineum. Dr. Frances Adams, of Syracuse, The truant school problem, and a paper by Dr. Abby Adams, of La Crosse, Wis., on Eclampsia was read. Dr. Dickinson also gave a paper before the league on Therapeutic application of electricity. Dr. Mann read a paper on Dress, illustrated by stereopticon views; Dr. Wende one on Skin diseases, and Dr. Gregory one on The pharmacopœia.

The first banquet of the league was held at The Genesee in June, 1895. The next was at The Niagara in June, 1897. In June, 1898, the banquet was again held at The Genesee, and at The Fillmore in June, 1899. The banquet was omitted in 1900 and in place of the June banquet, 1901, the league accepted an invitation by the Drs. Green, of Castile, to a delightful entertainment and collation there provided by them.

Instead of our prospective banquet in June, 1902, the league decided to entertain The Practitioners' Club, of Rochester, in October, 1901, which was accordingly done, holding a medical meeting in the Women's Building on Pan-American grounds, followed by a tea given by the Board of Women managers in the afternoon and our annual banquet in the evening at The Buckingham. The average yearly attendance of members since the league attained its present numbers is as follows: 1899-1900, 8 per meeting; 1900-1901, 10 and a fraction; 1901-1902, 11—showing a gradual increase in the attendance.

During the present year, 1901-1902, there have been 8 meetings of the league, 16 papers presented—10 by members and 6 by guests—and 2 informal talks—1 by a member and 1 by a guest.

The first meeting of the year was at the home of Dr. Jane W. Carroll, the second at the Woman's Building, Pan-American grounds; the third with Dr. Electa B. Whipple, the fourth with Dr. Helene Kuhlman, the fifth with Dr. Katharine Munhall, the sixth with Dr. Cora B. Lattin, the seventh with Dr. Lillian C. Randall and the eighth, with Dr. Jane N. Frear.

We have had 13 guests this year. The usual plan of each hostess serving refreshments was omitted (for the year) on trial and in compensation a midwinter picnic was held when "the flow of soul" was along the lines of pharmacy, for which Dr. Gregory was responsible.

The entertainment of The Practitioners' Club, of Rochester, with other invited guests was an enjoyable feature, if we may judge from the expressions of satisfaction which have reached us. The success was largely due to the helpfulness of the general committee—Drs. Carroll, Frye and Cummings—and to the table committee, Drs. Frye and Clayton.

The constitution of The Physicians' League has this year been revised and greatly improved, for which credit is due the committee, Drs. Denton, Randall and Carroll. We are indebted to the topic committee, Drs. Clayton and Frye, for systematically arranging the literary program, in assigning subjects of papers that were closely related for each evening's entertainment.

The league has unanimously voted to purchase and maintain a crib, for one year, in the new Crèche, which is established by the Western New York Branch of the Association of Collegiate Alumnæ. The decision was hastened by the work of the committee, Drs. Lattin, Huntley and Carroll. We have this year added one honorary member—Dr. Green, of Castile—to our roll and placed an honorary member—Dr. Mary Clayton—on the active list. The policy of this administration has been carried out with the greatest unanimity by the other members of the cabinet, Drs. Carroll and Kuhlmann.

From the epitomè presented, it must appear that some scientific work has been done and a moderate degree of success has been attained during the now closing decade. It may not be out of place to make a few recommendations: (1) we advise the consideration of making The Physicians' League a chartered society as is our neighbor, The Practitioners' Club; (2) we suggest that the league each year invite physicians, not members, to present papers, thus keeping ourselves in touch with others' work; (3) since our society is yet in the process of evolution let us not jeopardise its life by taking the league too seriously, but bear in mind that growth is the outcome of relaxation combined with hard work. If our shortcomings have been many, we at least lay claim to working in a spirit of honesty. We close the books at the end of this first decade for the opening of the new at the beginning of the second. May the records show greater progress in the coming years.

491 PORTER AVENUE.

